# Metal-on-Metal Hip Retrieval Analysis: A Case Report

**DOI:** 10.1155/2013/398973

**Published:** 2013-06-09

**Authors:** Thomas B. Pace, Kara A. Rusaw, Lawrence J. Minette, Brayton R. Shirley, Rebecca G. Snider, John D. DesJardins

**Affiliations:** ^1^Department of Orthopaedics, Greenville Health System, University of South Carolina SOM, 701 Grove Road, Greenville, SC 29605, USA; ^2^Department of Bioengineering, Clemson University, 301 Rhodes Engineering Center, Clemson, SC 29634, USA; ^3^Department of Pathology, Greenville Health System, Pathology Associates of Greenville PA, 8 Memorial Medical Court, Greenville, SC 29605, USA; ^4^Steadman Hawkins Clinic of the Carolinas, Greenville, 315 Medical Parkway, Suite 100, Greer, SC 29650, USA

## Abstract

This is a case report involving a single case with severe bone and soft tissue destruction in a young male patient with a 10-year-metal on-metal total hip arthroplasty. Following complete aseptic erosion of the affected hip greater trochanter and abductor muscles, the hip was revised for recurrent instability. Histological examination of the patient's periprosthetic tissues, serological studies, and review of recent medical reports of similar cases were used to support an explanation of the destructive process and better contribute to our understanding of human reaction to metal debris in some patients following metal-on-metal hip arthroplasty.

## 1. Introduction

Early metal-on-metal (MOM) hip implant designs were abandoned secondary to presumed insufficient material wear resistance and unacceptable high incidences of component loosening (67%) [1]. Subsequently, metal heads articulating with UHMWPE remained the implant selection of choice for most surgeons for the next 30 years. Osteolysis, aseptic loosening, and instability associated with ultrahigh molecular weight polyethylene (UHMWPE) wear debris led to a renewed interest in MOM prostheses in the 1990s and in the early part of the last decade. Changes in modern MOM hip prostheses including tighter manufacturing tolerances and improved metallurgy were proposed to eliminate the shortcomings and relatively high failure rates of earlier designs [2, 3]. This second generation MOM implant included the Metasul “polyethylene sandwich” acetabular cup insert with an embedded 28 mm metal articulating surface within the otherwise standard polyethylene cup insert. More recent changes in the use of metal-on-metal hip arthroplasty implants over the past decade have been wrought with new concerns of destructive tissue changes (pseudotumors) around the affected joint.

This report describes a case involving a dramatic relatively asymptomatic aseptic soft tissue and bony reaction in a second generation Metasul “polyethylene sandwich” type MOM total hip arthroplasty at ten years of followup. The patient was informed that the data from the case would be submitted for publication and provided written consent. 

## 2. Case Report

A 28-year-old male patient's right hip was primarily replaced in 1984 (surgery 1) due to joint degeneration secondary to developmental hip dysplasia and was then revised in 1996 for recurrent instability (surgery 2, Zimmer-Warsaw, IN) due to aseptic loosening and polyethylene wear. The right hip was revised again with an MOM 28 mm metal liner and matching head in 1999 (surgery 3, Sulzer Press Fit, Metasul Cup and Head) due to persistent instability. All surgical cultures confirmed aseptic loosening. 

The hip was clinically problem-free until 2009, when the patient suffered a dislocation while arising from bed. The patient denied antecedent pain prior to dislocation though he did have abductor muscle weakness and walked with a cane for two years prior to his presentation for dislocation. Treatment included a closed reduction and hip abductor brace; loss of greater trochanter was noted on radiographs at this time. Additional revision surgery (surgery 4: Zimmer, Epsilon Durasul constrained insert, and Cobalt Chrome 38 mm head) was required for recurrent instability 6 weeks later.

**Figure 1:**
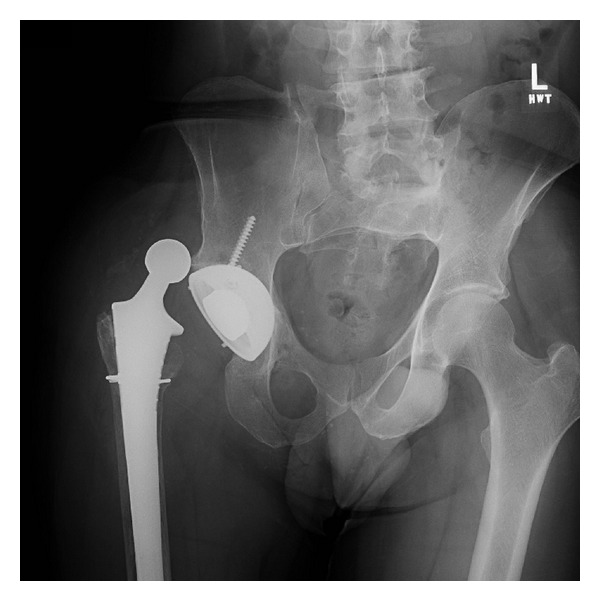
Ten year old hip with dislocation.

**Figure 2:**
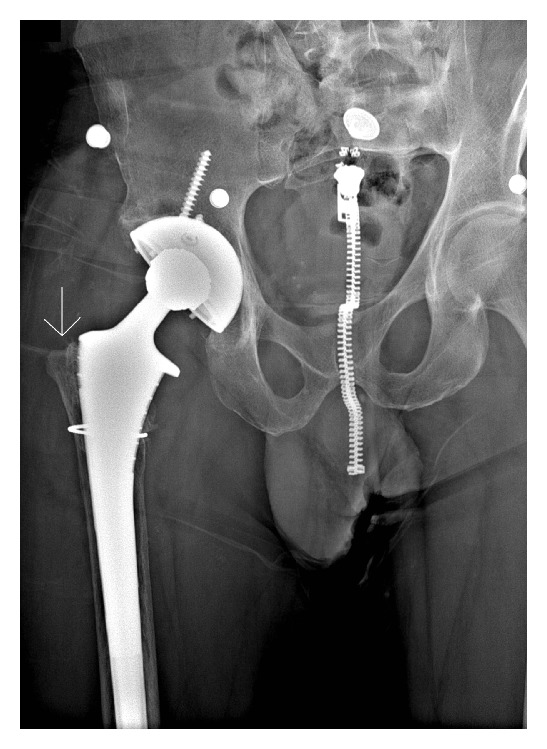
Status post reduction. Note GT bone loss (arrow).

Radiographs prior to surgery 4 of the hip 10 years after implantation showed total erosion of the greater trochanter. (Figures 1 and 2).

**Figure 3:**
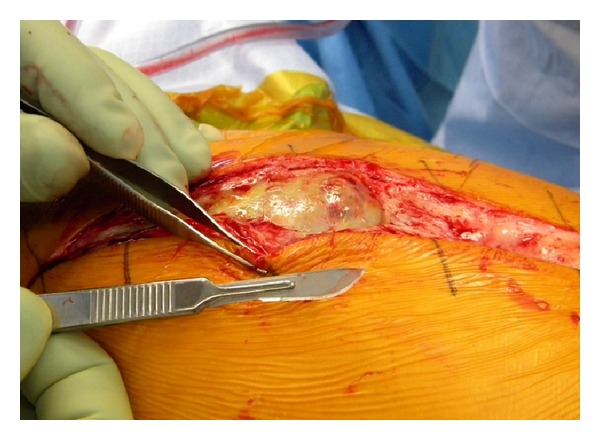
Revision surgery.

**Figure 4:**
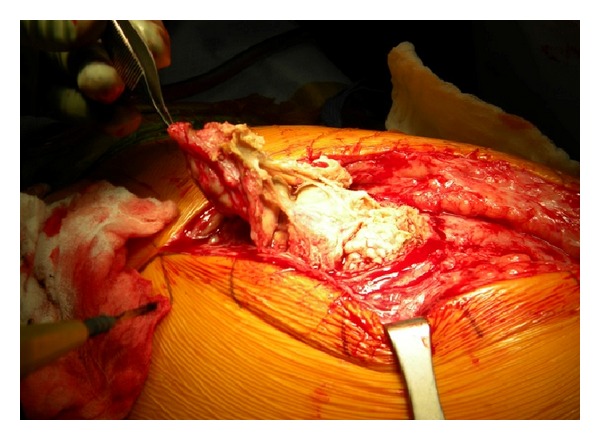
Revision surgery.

**Figure 5:**
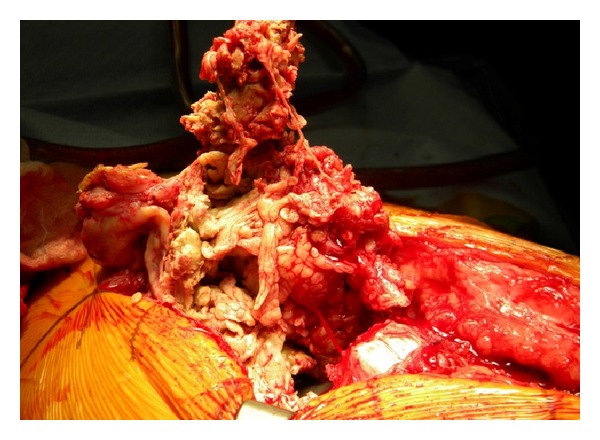
Revision surgery.

**Figure 6:**
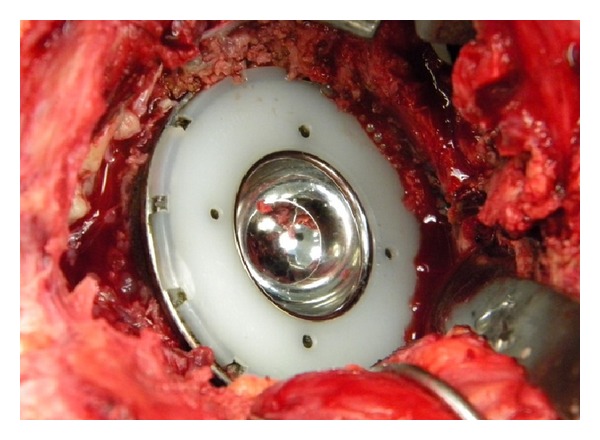
Metasul liner (removed).

**Figure 7:**
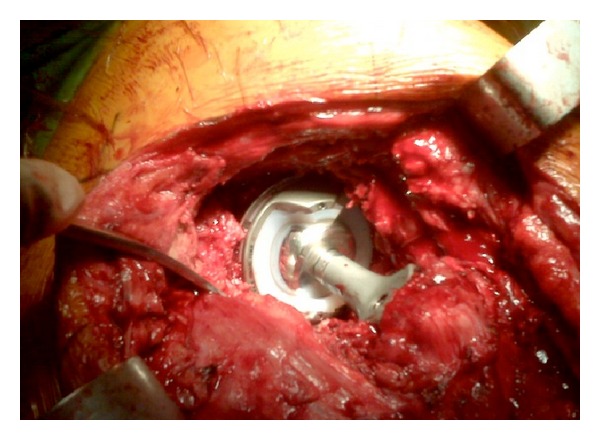
New exchanged constrained liner.

At the time of the final revision surgery, a large encapsulated tissue mass was found to incase the gluteus minimus, medius, and greater trochanter with amorphous aseptic necrotic tissue (Figures 3, 4, and 5). Complete excision of the 900 gram mass (18 × 15 × 6 cm) was performed. The acetabular shell was well fixated to the pelvis. The polyethylene liner locking mechanism was secure, and there was no evidence of backside wear of the polyethylene insert. The articular metal liner showed no gross adverse findings and no signs of impingement. Implant revision consisted of inserting an all-polyethylene constrained acetabular liner in the existing cup combined with a new 38 mm Cobalt-Chrome femoral head (Figures 6 and 7). The cup inclination angle was 60 degrees which was not felt to be ideal but was accepted as subsequent removal of the well-fixed cup may result in greater problems secondary to potential acetabular bone loss.

The right hip tissue cultures were negative for infection, and frozen tissue sections revealed no acute infection. Serologic laboratory values are shown in Table 1. The patient's serology showed leucopenia, eosinophilia, and high serum cobalt and chromium levels. Erythrocyte sedimentation rate was within normal values, while the c-reactive protein (CRP) was mildly elevated. Urine cobalt and chromium levels were also elevated (Table 2). 

**Figure 8:**
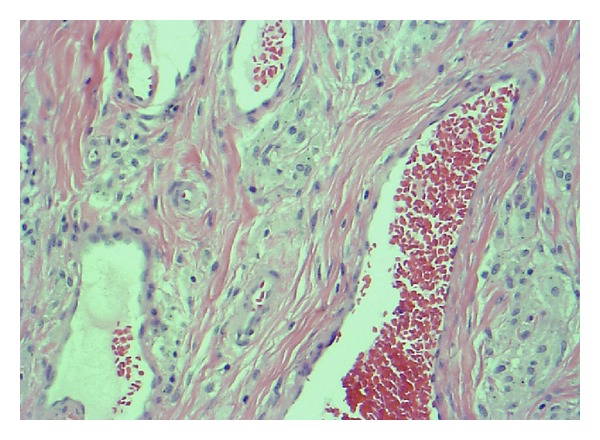
Pigment laden histiocytes not limited to vascular area.

**Figure 9:**
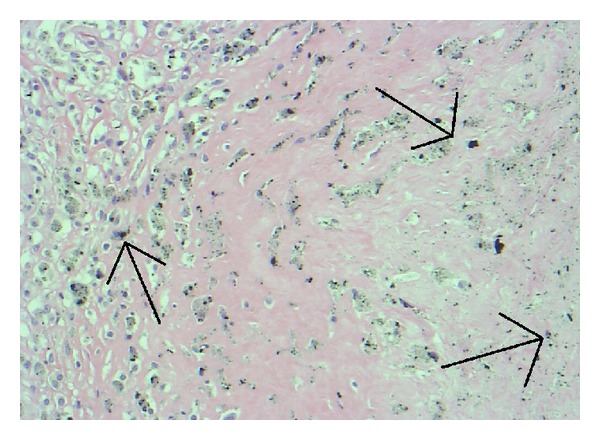
Histiocytes with black (metal) pigment (arrows) diffusely seen throughout tissue specimen.

Histological evaluation of the abnormal hip tissue revealed abundant tissue necrosis, fibrosis, and granulomatous inflammation with foreign body-type giant cell reaction (Figures 8 and 9). The foreign body-type macrophages contained foreign material suggestive of metallic particles. Aggregates of individual histiocytes with multiple foreign particles per cell were present. The particles ranged from round to thin and elongated in shape with most particles about 1 *μ*m in diameter. Scanning electron microscopy was not used to identify smaller particles. There was no bone, muscle, or tendon tissue present in the large mass excised. Scant presence of localized eosinophils with scattered histiocytes or small granuloma was found. Limited lymphocytes were identified, and there was no predilection for vascular areas.

## 3. Discussion

This case report highlights an atypical, largely asymptomatic process of extensive bone and soft tissue destruction in a patient with an MOM hip prosthesis 10 years after implantation.

First generation MOM implants used in the 1960s and 1970s such as the large diameter MOM hip implants had high failure rates of up to 15% at 12 years, 67% loose implants, and rapid decline at further followup despite reported wear rates of 0.003 mm/yr [1, 4, 5]. Proposed causes for their failure have included insufficient method of fixation (first generation cementing technique), implant impingement at the stem neck/cup border, and insufficient bearing material.

New concerns have been raised with newer generation MOM implants that were not reported in earlier designs. MOM prostheses have been shown to produce 40–100 times less volumetric wear than metal-on-polyethylene [6, 7]. The Metasul prosthesis design reported here has a published wear rate of 0.003–0.009 mm/yr versus 0.08 mm/yr for metal-on-polyethylene [5, 8]. However, MOM wear particles are very small so that a low wear volume can still produce a large number of biologically reactive particles that have been associated with tissue necrosis in other recent studies [6, 7, 9]. These clinical findings seem to be a marked change from the findings associated with failure of earlier MOM hip implants. 

Histological analysis in this case revealed differing stages of a histiocytic-based inflammatory process. Particulate laden histiocytes were present throughout the tissues and not localized to the vascular structures as seen in aseptic lymphocytic vasculitis-associated lesion (ALVAL) [10, 11].

The aggressive bone and soft tissue reaction seen here may well be an exaggerated macrophage and osteoclastic response to the metallic particulates smaller than the 1 micron size identified under routine light microscopy. Additionally, tissue necrosis is not typically seen in a response to wear (particles) from UHMWPE implants. The reaction is similar to a pseudotumor reaction as previously described by Clayton et al. in 2008 [12]. The early descriptions of pseudotumor formation in MOM hips described a histological pattern different from metal-on-UHMWPE hips predominately with the lack of plasma cells and a predominance of perivascular lymphocytes in the MOM hips [9, 10, 13]. There are other reports describing periprosthetic tissue eosinophilia [14] but none we are aware of that report serological eosinophilia associated with MOM pseudotumor type of tissue necrosis.

Reports have associated a higher incidence of pseudotumor formation in MOM hips with cup inclination angles >55 degrees [15]. The cup abduction angle in this patient's dysplastic hip measured 60 degrees. Edge loading of the bearing surfaces could have contributed to accelerated wear particle generation and subsequent exaggerated clinical response. The history of developmental hip dysplasia could have contributed to this patient's chronic hip instability. The greater trochanter on the contralateral hip in Figure 2 shows normal greater trochanteric development.

Hypersensitivity to metal implants has been a topic of concern for decades. Human immune responses to foreign materials are classified into five categories with metal-related implant reactions regarded as type IV, where macrophage activation over some period of time is the predominant feature [16]. However, most reports of metal hypersensitivity have less severe tissue reaction and histologically have high concentrations of lymphocytes and plasma cells that are not seen here [5].

High level of activity, unilateral disease, the number of preoperative hip operations, etiology (developmental hip dysplasia), and increased cup abduction have all been correlated with higher revision rates in metal-on-UHMWPE THA [17]. It is acceptable that particle-induced osteolysis is a shared adverse biological response to THA whether metal, ceramic, or polymer bearings surfaces are used. However, the more extensive soft tissue necrosis and bone destruction described in this case report appear to be a result of the cobalt-chromium wear particle toxicity from the MOM articulations. The 28 mm head size and previously stated risk factors may have contributed to increased metal wear and thus exaggerated the biological reaction. The presence of serological eosinophilia in association with a failed MOM THA in this report may indicate a more complex immune reaction than previously understood.

Many patients tolerate MOM hip arthroplasties, including this implant in particular, without problems [2]. However, this case demonstrates an insidious destructive tissue response in a relatively asymptomatic patient. The fact that this extensive tissue destruction was a silent, relatively painless process that did not prompt medical evaluation justifies close clinical followup of even minimally symptomatic patients. 

## Figures and Tables

**Table 1:** Patient's serology laboratory values.

WBC count: (Ref. range: 4.8–10.8) TH/mm^3^	3.1 TH/mm^3^ (Low)
Neutrophil ABSOL: (Ref. range: 1.4–6.6) TH/mm^3^	1.10 TH/mm^3^ (Low)
Lymphocyte ABSOL: (Ref. range: 1.0–3.5) TH/mm^3^	0.60 TH/mm^3^ (Low)
Mononuclear: (Ref. range: 3–12) %	19.8% (High)
Eosinophil: (Ref. range: 0–4) %	9.2% (High)
Hemoglobin: (Ref. range: 13.5–17.5) g/dL	13.7 g/dL
SED rate: (Ref. range: 0–15) mm/hr	6 mm/hr
ALK phosphatase: (Ref. range: 32–91) IU/L	171 IU/L (High)
CRP noncardiac: (Ref. range: <3.0) mg/L	10.8 mg/L (High)

**Table 2:** Cobalt and chromium levels.

	Urine studies	Serum studies
Cobalt levels (Ref. range)	9.5 (<2.8 mcg/L)	8.4 (<1.8 mcg/L)
Chromium levels (Ref. range)	12.0 (<2 ng/mL)	8.8 (>1.4 mcg/L)
